# Fiber Bragg Grating Dynamic Sensing Through a Dispersive Spectrometer

**DOI:** 10.3390/s26134152

**Published:** 2026-07-01

**Authors:** Yohan Barbarin, Alexandre Lefrançois, Victor Colas, Sylvain Magne, Thomas Blanchet, Laurent Fieschi, Vincent Chuzeville, Jean-Marc Chevalier, Jérôme Luc, Antoine Osmont

**Affiliations:** 1 CEA, DAM, GRAMAT, BP 80200, 46500 Gramat, France; alexandre.lefrancois@cea.fr (A.L.); victor.colas@univ-lorraine.fr (V.C.); jerome.luc@cea.fr (J.L.); antoine.osmont@cea.fr (A.O.); 2 Université Paris-Saclay, CEA, List, 91120 Palaiseau, France; sylvain.magne@cea.fr (S.M.); thomas.blanchet@cea.fr (T.B.); 3 CEA, DAM, CESTA, 33116 Le Barp, France

**Keywords:** fiber Bragg grating, energetic material, detonation, deflagration, strain, pressure

## Abstract

In the field of shock physics and energetic materials, Fiber Bragg Gratings (FBGs) are used to measure shock velocity, detonation velocity and shock pressure levels. They are also used to measure strain in structures loaded with explosive effects. FBG sensors are known to be light, small, immune electromagnetic environments and have fast response compared to electrical sensors. To use one or more gratings along a fiber, a high-resolution spectrometer with a high sampling rate has been developed. This dynamic spectrometer employs time-multiplexing by wavelength-to-time conversion using dispersion. It provides a complete view of the spectra evolution at a rate of 100 MHz. Thus, complex phenomena can be observed. In this paper, the interrogation technique is presented in more detail, and experimental results are discussed. The experiments presented are a low-pressure shock velocity measurement in epoxy, a deflagration-to-detonation transition in a porous energetic material, a Shock-to-Detonation Transition in a dense energetic material, a tentative-to-measure pressure level in epoxy from an FBG made in a sapphire fiber and multi-point strain measurements up to eight FBGs. The advantages and limits are discussed for each type of experiment.

## 1. Introduction

In the field of shock physics and energetic materials, experimental parameters are required to improve Equations of States (EOSs) and to run complex hydrodynamic simulations. Transient physics, like a Shock-to-Detonation Transition (SDT), is of great interest. The main physical quantities ideally recorded continuously during such a fast experiment are shock position and velocity, detonation velocity, and possibly longitudinal stress profile. The in situ shock and detonation velocities are very valuable inputs. Typical velocities are in the range of a few km/s. The acquisition time lasts from a few μs to tens of μs at a rate of 100 MHz or higher. These physical measures obviously need to be experimentally acquired with minimum intrusiveness. Classically, electrical measurements are used. For detonation velocity and detonation wave profile measurements, Electrical Shorting Pins (ESPs) are commonly used [1]. This is a discrete chronometric measurement; the pin diameter is ~1 mm, and it is rarely implanted into High Explosives (HEs). The response time of such sensors can be below 5 ns, however. Pressure levels are measured electrically at a few points with thin-film gauges [2]. The pressure gauge material can be adapted to cover various ranges, i.e., carbon [0–4] GPa, and manganin [0–100] GPa. The response time of such gauges depends on their thickness and can be as fast as 50 ns for 50 μm thick gauges. The main drawbacks of this technique are that pressure gauges are sensible to electromagnetic (EM) fields, and that their footprint of a few mm^2^ is relatively intrusive. Shock velocities can be extracted by monitoring surfaces using optical interferometry [3]. Millimeter waves [4,5] are also used to measure inside the materials, but the material permittivity needs to be known and the measurement is averaged radially over a few millimeters depending on the collimator design involved.

New materials, alloys or assemblies are regularly characterized by shock loading to adjust Equations of States (EOSs) and elastic–plastic behavior parameters. The structural response is typically measured using strain gauges like in aircrafts [6]. Electrical Strain Gauges are a few mm^2^ in area and need individual electrical connections. These types of sensors offer a satisfactory response time (~1 µs), but remain relatively intrusive and are also sensitive to electromagnetic perturbations. Energetic materials are used to create fast and high-intensity loading on structures for characterization, and explosives are typically initiated by a detonator which is itself ignited by a high-voltage burst.

In this paper we show that Fiber Bragg Grating (FBG) can also be used to measure these physical values with low intrusiveness and EM immunity. FBGs can be as thin as 40 μm in diameter and have been used for decades to assess strain in structural health monitoring (SHM). Commercial interrogators typically monitor many peak wavelengths at a rate of a few kHz, and very rarely above 100 kHz. In shock experiments, the spectra acquisition rate must be at least two orders of magnitude higher (>10 MHz) and it is sometimes necessary to record the evolution of all spectra and not only the wavelength peaks.

For shock sensing, Chirped Fiber Bragg Grating (CFBG) is used. Our paper from 2020 [7] explains the concept in detail and presents many experimental results. The FBG can be as long as 100 mm, and chirping provides a wide spectrum (20–30 nm). If the CFBG is progressively destroyed by the shock or detonation wave, its spectral width decreases accordingly. The straightforward measurement consists of recording the reflected intensity of the CFBG. With accurate calibration, this intensity is correlated to the shock or detonation velocity. The measurement strongly depends on the effective shortening of the CFBG. If the pressure level is insufficient, the measurement does not work. Polymer CFBGs are being developed to increase the sensitivity [8,9], but their length is currently limited to ~20 mm. Another solution presented in this paper is to record the entire spectra, but at a high sampling rate.

A 100 MHz dispersive spectrometer is presented and has been evaluated in different experiments. This acquisition rate is about one thousand times faster than commercial products. This concept is known under different names: Time Domain Optical Sensing [10], Photonic Time-Stretch [11] or Time-Stretch Dispersive Fourier Transform [12]. It was applied to FBG in 2010 by Xia et al. [13] and in 2011 by Chao et al. [14]. To our knowledge, only Rodriguez et al. have applied this concept for the dynamic measurement of an FBG spectrum [15,16] in shock physics applications.

The experiments presented in this paper have different timeframes. The slowest phenomena last 1.6 ms; it is a multi-point strain measurement of up to eight FBGs on a structured loading by thin explosives. The deflagration-to-detonation transition in a porous energetic material presented lasted about 60 µs. Finally, the low-pressure shock velocity measurement in epoxy material, Shock-to-Detonation Transition in a dense energetic material and tentative-to-measure pressure level in epoxy from an FBG made in a sapphire fiber are recorded within 5 µs.

## 2. Dispersive Dynamic Spectrometer

**Figure 1:**
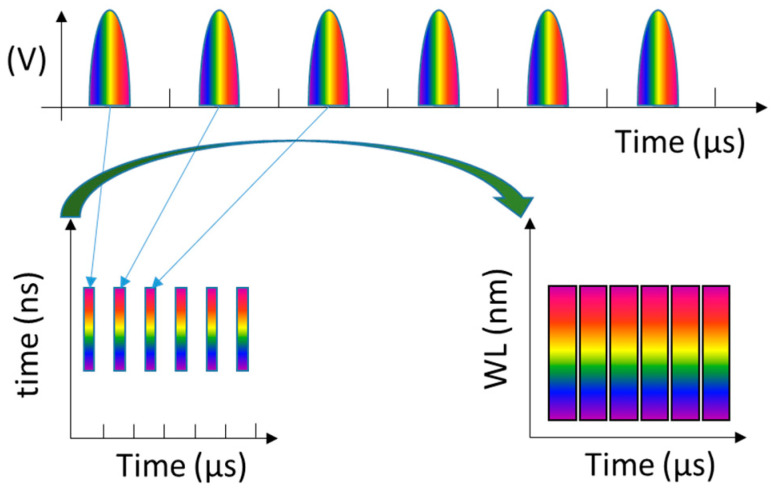
Time domain multiplexing of the dispersive spectrometer. The demultiplexing is done numerically.

**Figure 2:**
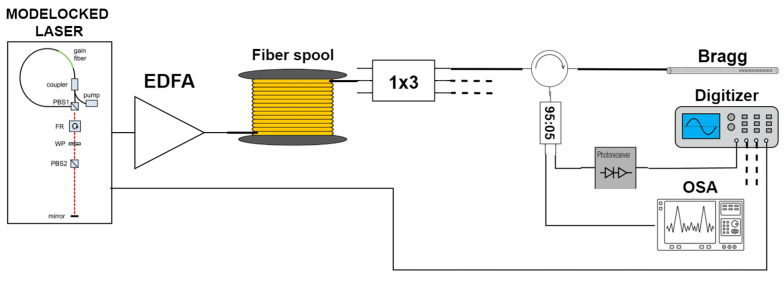
Schematic representation of the dispersive spectrometer with 3 measurement channels.

The 100 MHz dispersive spectrometer uses chromatic dispersion instead of a diffraction grating or prism to separate wavelengths. This enables real-time analysis of optical spectra. The optical spectra are multiplexed in time as illustrated in Figure 1. For an active measurement (i.e., to interrogate an FBG), a pulsed optical source with a wide spectrum is required. The optical source used here is a mode-locked fiber laser [17] (Menlo Systems C-Fiber or Menlo Systems ELMO), which provides a broad spectrum (~80 nm, centered around 1560 nm) to probe the FBG. The laser’s repetition rate (100 MHz) sets the acquisition frequency of the spectra. The spectrum is temporally stretched using the chromatic dispersion of a long single-mode optical fiber (several km). A fiber 5 to 10 times shorter and highly dispersive [18] could be used, but optical losses would be in the same range. Finally, the optical signals are electrically converted and amplified by photoreceivers (New-focus 1474-A). In LANL publications [15,16], wavelength-to-time conversion occurs after FBG reflection. We propose to do it differently for two reasons. In the system presented in Figure 2, we first convert the wavelength into the time domain, making it possible to interrogate three optical fibers simultaneously using the same laser source and without multiplying the spools of several kilometers of dispersive fiber. This approach looks like an ultrafast tunable laser interrogator. Furthermore, the chronometry is also simplified, since a fiber spool with many km would create a significant delay which may slightly vary with temperature. In our approach, the reference time is given by the digitizer which is triggered by the experiment and the optical distance between the sensor and the detector. The drawback is that the averaged power of the laser should be carefully adjusted to avoid self-phase modulation in the long fiber, which could be created by the pulse’s high peak intensity [19]. The laser spectrum should remain the same before and after the fiber spool.

In all cases, the length of this fiber and its dispersion coefficient define the spectrometer’s span. The dispersion *D*(*λ*) of a standard SMF28 single-mode fiber is commonly expressed by Equation (1), which combines the dispersion of silica (*S*_0_ = 0.090 ps·km^−1^·nm^−1^) and the guided mode dispersion in the single-mode fiber (*λ*_0_ = 1310 nm).

(1) $$D\left(\lambda \right)=\frac{S_{0}}{4}\left(\lambda -\frac{\lambda_{0}^{4}}{\lambda^{3}}\right)\;ps\cdot km^{-1}\cdot nm^{-1}$$

The spectrometer’s span can be estimated by rewriting the equation and setting the fiber length *L* and the time period *T* between two laser pulses.

(2) $$Span\approx \frac{4\cdot T}{S_{0}\cdot L}{\left(\lambda_{c}-\frac{\lambda_{0}^{4}}{{\lambda_{c}}^{3}}\right)}^{-1}\;nm$$

At 100 MHz, the period *T* is 10 ns. Using 10 km of optical fiber and a pulsed source with a central wavelength *λ_c_* of 1560 nm, the resulting span is approximately 57 nm. Achieving excellent spectral resolution over this temporal range requires an ultrafast acquisition system. Our most recent digitizer (Keysight UXR0204A) has a 20-GHz bandwidth and, more importantly, a sampling rate of 128 GS/s per channel, providing 1280 measurement points per spectrum. This number of measurement points is twice that of a compact CCD-based spectrometer, which typically has 512 pixels [20]. With such a high digitizer sampling rate, the spectral resolution—without any signal processing—is approximately 0.045 nm for 10 km of fiber. The spectral resolution can be further improved by reducing the spectral measurement range. For example, with 30 km of fiber, the span is about 20 nm and the spectral resolution is 16 pm. While this spectral range is much narrower than the source spectrum (~80 nm), this is not an issue because the unnecessary wavelengths are effectively filtered out by the FBG. Since the FBG is used in reflection, non-reflected wavelengths are lost at the fiber end, preventing spectral overlap between consecutive laser pulses.

A single digitizer has the advantage of recording the spectra of three or even four optical fibers, each containing one or more FBGs or CFBGs. To perform temporal demultiplexing of the spectral signals, it is more convenient to record the pulsed laser’s clock signal on one of the digitizer channels. The laser’s repetition rate can also be extracted from the initial moments when the FBGs have not yet experienced any spectral shift. However, this approach is challenging for long-duration signals, as the required precision is in the order of one time step (7.8 ps) over the entire signal duration. Over a 100 μs period, the laser’s repetition rate (~100 MHz) must be known with an accuracy better than ±7.8 Hz; otherwise, a non-negligible temporal drift will appear in the spectrogram, affecting the measurements. In the system shown in Figure 2, one channel is dedicated to the pulsed laser’s clock signal.

**Figure 3:**
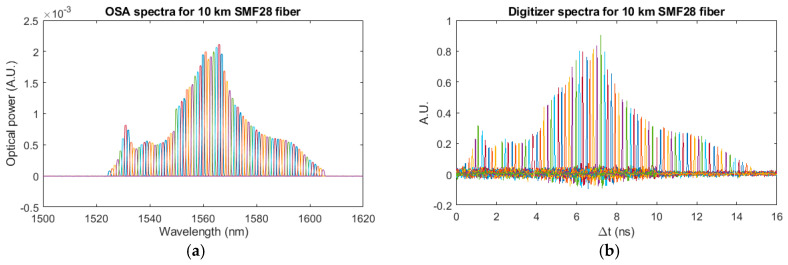
Measured spectra (optical (**a**) and temporal (**b**)) to obtain the wavelength-to-time calibration curve. The wavelength is varied with a tunable filter and a fibered reflector. The mode-locked laser used was a C-Fiber from Menlo Systems.

The adjustment of the wavelength-to-time conversion is the most crucial part of the calibration. It can be performed simultaneously on all three channels. A narrow tunable optical filter is inserted immediately after the dispersion stage, and the FBGs are replaced with fiber mirrors covering all wavelengths. The optical path length is also constant, and no additional time corrections are needed. Reference optical spectra are recorded using a calibrated Optical Spectrum Analyzer (OSA). A comparison of the source spectrum slices for a 10 km fiber length is presented in Figure 3 for the C-Fiber mode-locked laser. The source shape is preserved, and the wavelength-to-time relationship appears highly linear. Similar results are obtained with the ELMO mode-locked laser: only the optical spectrum shape is smoother.

**Figure 4:**
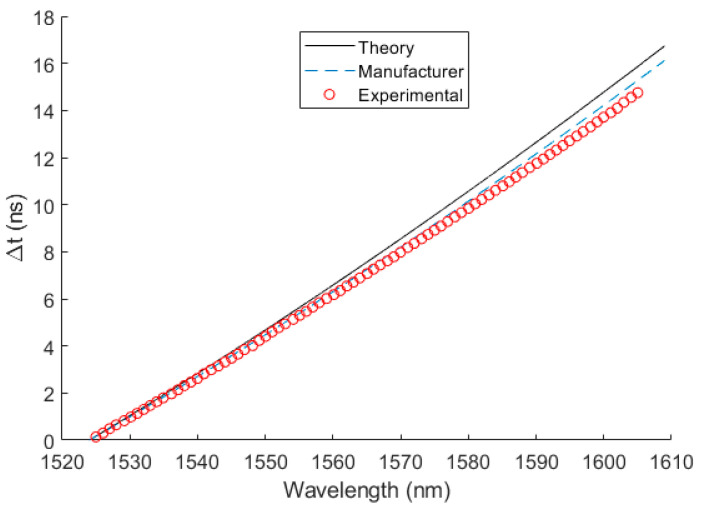
Wavelength-to-time curves with 10 km of single-mode fiber.

This is confirmed by the wavelength-to-time curve shown in Figure 4. The curve is compared with both theoretical predictions and the manufacturer’s model. The experimental curves obtained for the various configurations of the dynamic spectrometer are used during signal processing to convert the time axis (0–10 ns) between two pulses into the wavelength domain. A reference spectrum is recorded just before each test for each fiber using an OSA, allowing the spectra to be realigned by detecting, in the time domain, the position of a first narrow peak (FBG) or the edge (at half-maximum) of a broader spectrum (CFBG). Indeed, depending on the length of the optical fiber between the spectrometer system and the Bragg grating, the time axis in Figure 4 can be freely offset.

The wavelength-to-time conversion has to be further corrected regarding the physical properties of the FBG line. For instance, if many short FBGs are spaced physically apart by 25 mm, the additional time is twice this distance multiplied by the fiber effective index (245 ps). With eight FBGs, the additional time between the last FBG and the first one would be 1.71 ns, which is about 20% of the timeframe. This aspect has to be taken into account when designing a chain of FBGs. Sometimes it is more interesting to sort the wavelengths in reverse order. A similar effect occurs with a CFBG. The spectrum is spread in the time domain according to the dispersion and, additionally, the time of flight between the start and the end of the grating. Our longest CFBG is 100 mm long, and the additional time on the digitizer would be 0.98 ns.

The temperature of the long single-mode fiber may influence the wavelength-to-time curves. In our case, the measurement system is placed in a measurement room with stabilized temperature. The fiber spools are isolated from the laser and the digitizer so no additional heating is provided to them. In the worst case, the temperature of a fully loaded measurement room might rise from 22 °C to 30 °C. It would rarely go below 20 °C. In [21], a value of 2.1 × 10^−6^ ps·nm^−2^·km^−1^·°C^−1^ for the single-mode fiber dispersion slope ∂*S*/∂*T* Equation (3). For L = 18 km and Δ*T* = 10 °C, the dispersion change is about 0.89 ps/nm, which is a change of only 0.03%.

(3) $$∆D=\frac{\partial S}{\partial T}\;\left(\lambda -\lambda_{0}\right)\cdot ∆T$$

## 3. Experimental Results

### 3.1. Low-Pressure Shock Velocity in Epoxy

Chirped FBGs have been used to measure shock and detonation in dense materials by measuring the reflected intensity. If the CFBG is not continuously broken during the shock propagation, the grating length is not reduced and the measurement is not relevant. Using the dynamic dispersive spectrometer, the shock into the target can be tracked with high resolution. In this subsection, we present a shock velocity measurement in an epoxy target plate. The experiment is a symmetric impact of 20 mm thick epoxy plates. It was performed in a single-stage gas gun facility 35 mm in diameter. More details on the setup can be found in [22], where pressure measurement was investigated with two FBG orientations.

To avoid additional strain, the epoxy target was not molded around the CFBG. The target was first molded in a cylinder shape without any fiber and then machined to obtain good surface quality. Finally, a 1 mm diameter hole was drilled in the center of the target. Epoxy was again filled into the hole and the CFBG was placed. The CFBG has a polyimide coating and is 250 µm in diameter, and has been glued with the same epoxy around the fiber. The CFBG is 18 mm long and has a chirp rate of 1.461 nm/mm, which gives an initial spectrum width of 26.3 nm. The characterization by optical frequency-domain reflectometry (OFDR) can be found in [7]. The short wavelengths start at the fiber tip and will experience the shock first. For this experiment, a 10.6 km long fiber spool was used in the dispersion stage.

**Figure 5:**
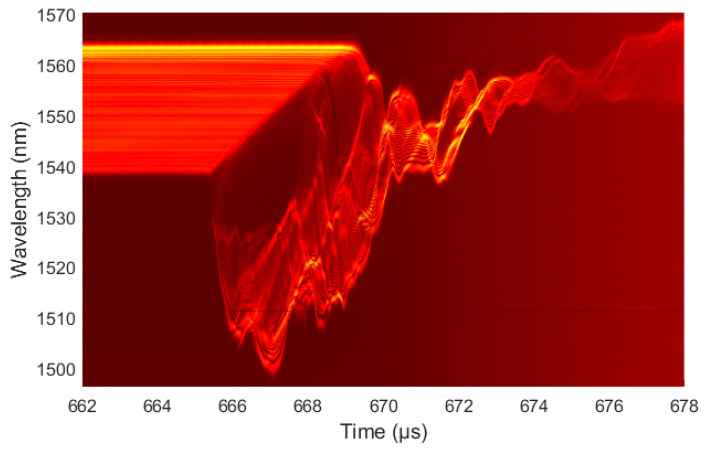
Spectrogram of a 20 mm long CFBG during a shock through an epoxy target. The color map on the *Z*-axis is with a linear scale. A total of 10.6 km of SMF was used.

The impactor was launched at 293.8 m/s onto the target. The time series recorded was synchronized with the laser clock. Then, the corresponding wavelength-to-time calibration curve was used to finally obtain the spectrogram plotted in Figure 5. The spectrogram shown has a trapezoidal shape; the visible slope corresponds to the shock position along the CFBG. The spectrogram is however richer with wavelength downshifts. The initial wavelength shift at 666 µs is about −25 nm, and the maximum at 667 µs is about −35 nm. In fact, the shock coupling from the target into the silica fiber is not instantaneous with this fiber orientation [22], and it gets worse with a high shock impedance mismatch like exists here (epoxy versus silica). We were able to obtain a first detailed modeling between silica and aluminum [23], and it can be further improved by separately taking into account the longitudinal and radial strains of the grating. Here, assuming that the CFBG is not shortened, the observed delay of the shock to the CFBG’s core is about 300 ns, which is significant. The averaged wavelength shift is about −27 nm, which would correspond to a pressure level of about 8.4 kbar. Again, this setup and CFBG are not ideal for in situ pressure measurements. We would recommend multiple FBGs oriented parallel to the shock wave even if the fiber exit has to be protected.

**Figure 6:**
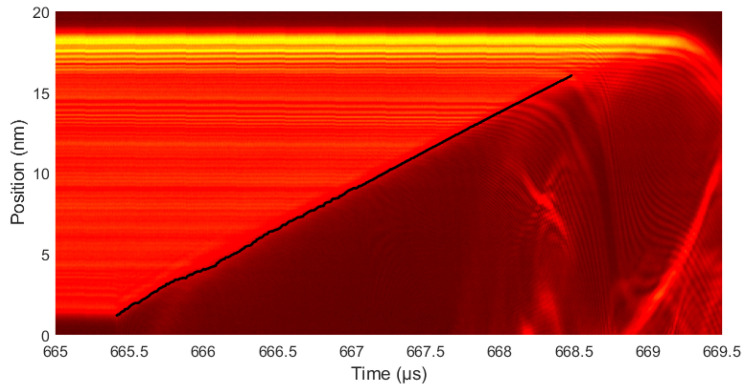
Shock X-T diagram in an epoxy target retrieved from the spectrogram in Figure 5.

The relevant information in this spectrogram is the shock wave position as a function of time. Since the CFBG is not progressively shortened by the shock wave, an intensity measurement like in [7] would not have worked. However, with the dispersive spectrometer, almost the complete spectrogram allows for tracking of the CFBG edge and therefore the shock position as a function of time (X-T diagram). The spectrogram was post-processed: the *Y*-axis is converted into millimeters, and the CFBG edge between 665.5 and 668.5 µs is detected. The X-T diagram is plotted in Figure 6 on top of the spectrogram. The averaged slope gives a shock velocity of 4808 ± 17 m/s.

This is an interesting demonstration of the measurement system because the shock position could be tracked continuously within the target along the CFBG even though the grating is not continuously damaged. We can also model the pressure level and estimated it. This experiment was for a sustained shock, but the measurement can be used for decaying shocks.

### 3.2. Shock-to-Detonation Transition

At the Gramat CEA research center, highly explosive compounds are evaluated. To obtain the kinetic parameters, Shock-to-Detonation Transition (SDT) experiments are performed with different sustained and/or unsustained shock levels. This depends on the impactor material, thickness and velocity at the impact. The relevant physical quantities that could be obtained from a CFBG are the shock velocity, the steady-state detonation velocity and the run distance (or time-to-detonation) [24,25]. The detonation destroys the CFBG efficiently and progressively, but this is not always the case in the shock part. We have often observed discrepancies between the ESP and CFBG data. As mentioned above, we are evaluating the use of polymer CFBGs instead of silica [8,9], but they are currently not fully operational and are limited in length (~20 mm). The dispersive spectrometer gives a complete in situ vision of the experiment. To illustrate this, the following experiment is presented. The material compound under study is HMX 90% and 10% binder in weight. The explosive has a wedge shape with a 30° angle to place at different-height ESPs and to avoid any back release wave. In the center, a 0.5 mm hole is drilled to place the same type of CFBG as in the previous experiment (18 mm long). This setup was placed at the end of a 98 mm diameter powder gun. The impactor was made of aluminum 6061T6 (15 mm thick). The measured impact velocity was 1408 m/s.

**Figure 7:**
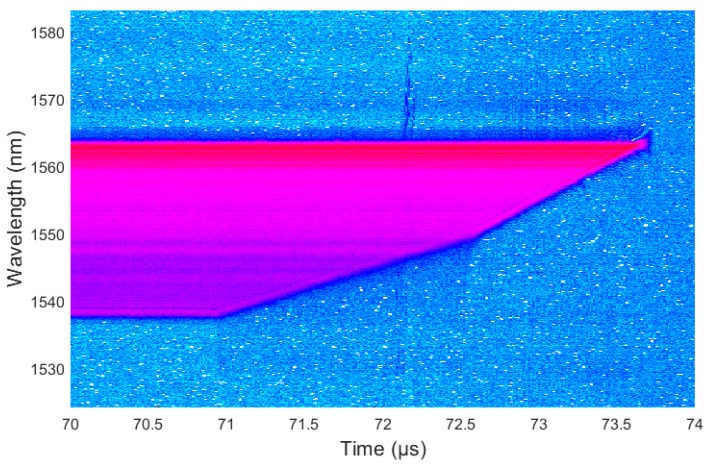
Spectrogram of an 18 mm long CFBG during an SDT. The color map on the *Z*-axis is with a log scale to highlight the edges. A total of 18 km of SMF was used.

The processed spectrogram is plotted in Figure 7. In this experiment, the pressure level in the shock part (between 70.9 and 72.6 µs) was obviously sufficient to progressively extinguish the CFBG, because there is not any signal below the first slope. Signal processing more easily detects the CFBG edge corresponding to the shock and detonation waves.

**Figure 8:**
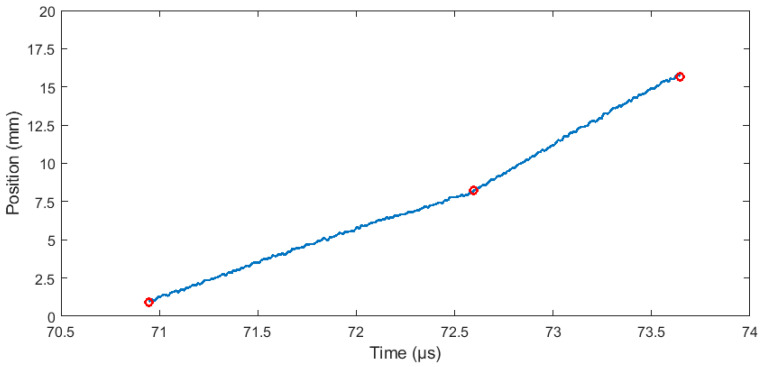
X-T diagram during an SDT. The red circle are the limits used for the linear fits to get the shock and steady detonation values.

By converting the *Y*-axis into millimeters, the X-T diagram is obtained and plotted in Figure 8. Two slopes are clearly visible. The linear fits between 60.9 µs, 72.7 µs and 73.7 µs gives a shock velocity of 4352 ± 14 m/s and a steady detonation velocity of 7340 ± 25 m/s.

Many SDT experiments were not well tracked in the shock zone using the system based on the amplitude measurement [7]. The CFBG is not certainly always damaged, which affects the continuous measurement. The dispersive spectrometer overcomes this issue and provides almost the same number of points in the X-T-diagram (100 MHz versus 250 MHz). Since long CFBG can be realized (>100 mm), a setup with a succession of explosives could be characterized.

### 3.3. Deflagration-to-Detonation Transition in a Low-Density Explosive

The deflagration-to-detonation transition (DDT) in energetic materials is still far from being understood despite the studies made in the 1970s [26,27]. This is due to the fact that the mechanisms involved are multiphysics, multiphase, and multi-scale. In order to progress in DDT modeling, we have improved our experimental setup previously used for the dynamic compaction of explosive powders [28]. In this subsection, a low-density explosive compressed at 80% of the theoretical maximum density is tested. It is composed of 96% HMX (octogen) and 4% fluorine rubber (Viton). The material was first machined into a 70 mm diameter and 57 mm high cylinder. Then, one side was machined again to obtain a flat surface area of 50 × 57 mm^2^. The explosive was inserted into a metallic casing in contact with a piston at one of its ends. A transparent polycarbonate window was finally positioned at the flat surface of the setup. This window allows for stereo-correlation fast imaging. Two 50 mm long CFBGs were also inserted between the explosive and the polycarbonate element.

**Figure 9:**
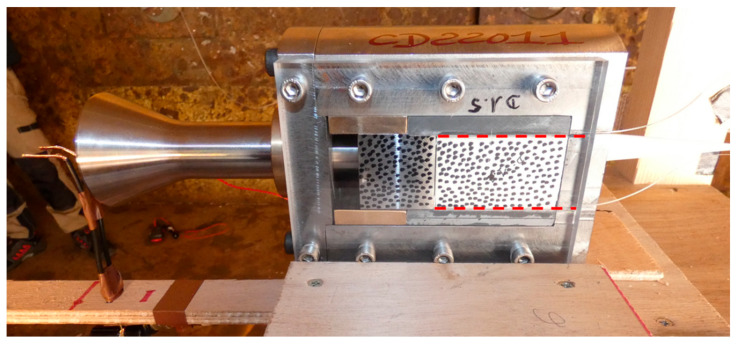
Photograph of the DDT test setup. CFBGs are represented by two red dashed lines.

The experiment consists of impacting one end of the piston with a projectile launched by a gas gun. The piston then undergoes translational motion and mechanically loads the explosive charge. Compaction waves propagate through the latter and coalesce until, under certain conditions, they initiate the material in an intermediate regimen. The measurement system used allows for tracking of the intensity and chronometry of the different phenomena (compaction rate, initiation distance, reaction propagation, etc.). A photograph of the setup is provided in Figure 9. The piston, as well as the explosive, are dotted to allow optical tracking by stereo-correlation. CFBGs are represented by the two red dashed lines. For the following experimental result, a projectile was launched at 253 m/s onto the left side of the piston. The measurement was triggered upon contact between the projectile and the piston.

**Figure 10:**
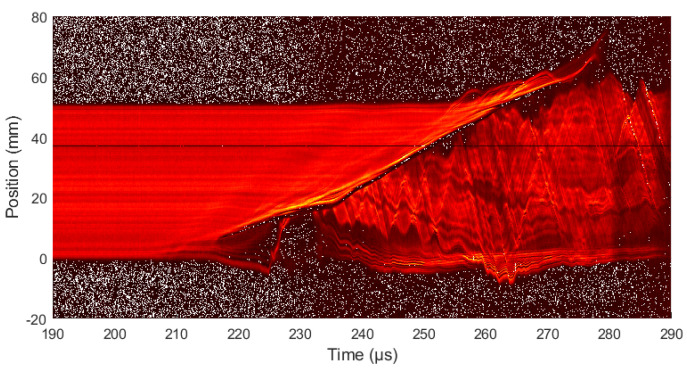
Spectrogram of a 50 mm long CFBG during a DDT experiment.

The spectrogram of the CFBG located on the lower part of the setup is shown in Figure 10. It was processed in the same way as in the two previous experiments presented above. The *Z*-axis, with the color map, is in log-scale to highlight the CFBG edges. The CFBG is not continuously destroyed during compaction; therefore, such a spectrogram can provide an estimate of the timing of both the compression and reactive phases. A first signal propagating at a velocity of about 570 m/s is visible between 215 µs and 235 µs. This phenomenon corresponds to the compaction wave propagating through the explosive. These data are in agreement with the video observations. At 235 µs, an abrupt change in the slope with a noticeable acceleration of the phenomenon is measured. The velocity is about 1100 m/s. This second wave is associated with a reactive phenomenon propagating through the charge.

These results are particularly interesting, as they represent the first measurement of an intermediate deflagration-type reaction for this configuration. The CFBG thus offers an in situ measurement of the material’s behavior under low shock, and helps to overcome the optical limitations associated with stereo-correlation fast imaging. No other metrology was successful in characterizing such a DDT experiment.

### 3.4. Sapphire FBG for High Pressure Measurements

**Figure 11:**
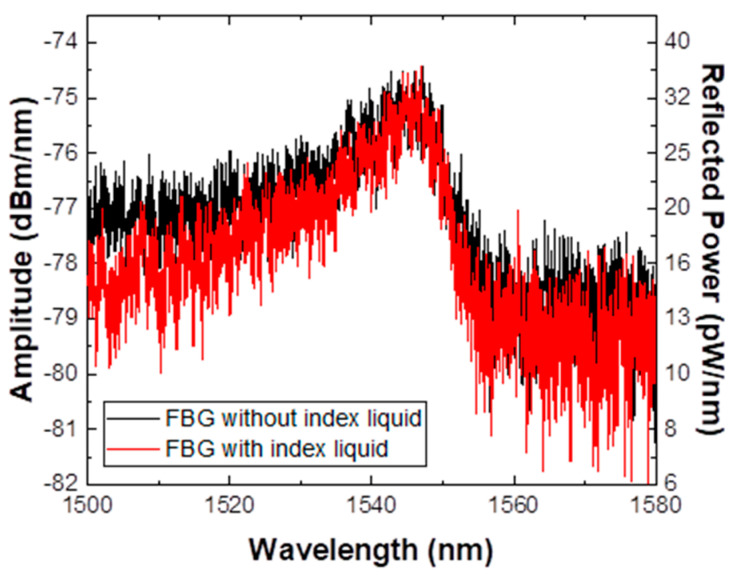
Reflected spectrum of the FBG on a multimode sapphire fiber.

Following our work on high-pressure measurements with silica FBGs and CFBGs [22,23], we have investigated the use of sapphire FBGs to extend the pressure range. Silica gratings are limited to about 60 kbar, while sapphire FBGs would, in theory, be able to measure pressure up to 200 kbar. Since sapphire FBGs are not commercially available, the first step was to fabricate them. The gratings have been photo-inscribed on the CEA List (FemtoBragg platform) involving a 100 µm diameter sapphire fiber using the phase mask technique and an Astrella (Coherent) fs-laser (Ti:Sa) emitting around 800 nm with a pulse duration of 35 fs at a maximal energy of 7 W. More details on the fabrication process can be found in [29]. These FBGs were also tested for high-temperature measurements up to 1500 °C for several hours, where no significant Bragg wavelength shift was found [30]. Due to the high multimodal behavior of the sapphire fiber, the Bragg peak is wider than the typical one inscribed in silica SMF. In the following experiment, the FBG was engraved at the end of a 10 cm long sapphire fiber. The reflected spectra were recorded with and without index liquid at the unconnected fiber extremity, with plotting in Figure 11 before integration. The overall shape is slightly triangular, which is typical for a grating inscribed in a multimode fiber [29].

**Figure 12:**
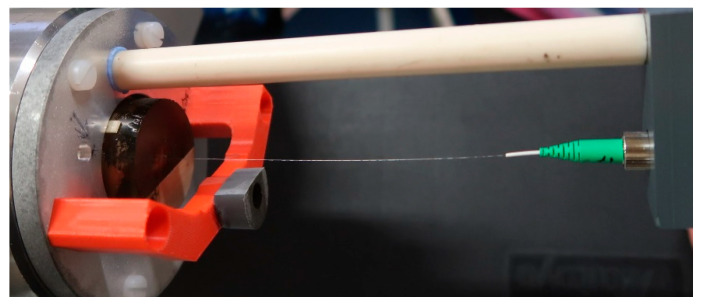
Photograph of the sapphire fiber exiting the epoxy target at the end of the gas launcher.

This short sapphire fiber was carefully glued onto a PMMA plate. Since this fiber is multimodal, the fiber is kept straight to try to get the maximum optical power into the fundamental traverse mode. A photograph of the setup at the end of the gas launcher is shown in Figure 12. Because of the not very reflective grating (compared to the one inscribed in silica SM fiber) and the additional coupling losses between the silica SMF and the sapphire multimode fiber (100 µm sapphire fiber core), the laser in the dispersive spectrometer was used for only one channel instead of three. Furthermore, the signal was amplified by an EDFA before detection. The experiment involved a symmetric impact with the PMMA impactor and target. It was done at a low pressure level (~4.2 kbar) similar to that presented in Section 3.1. The measured impact velocity was 291.7 m/s.

**Figure 13:**
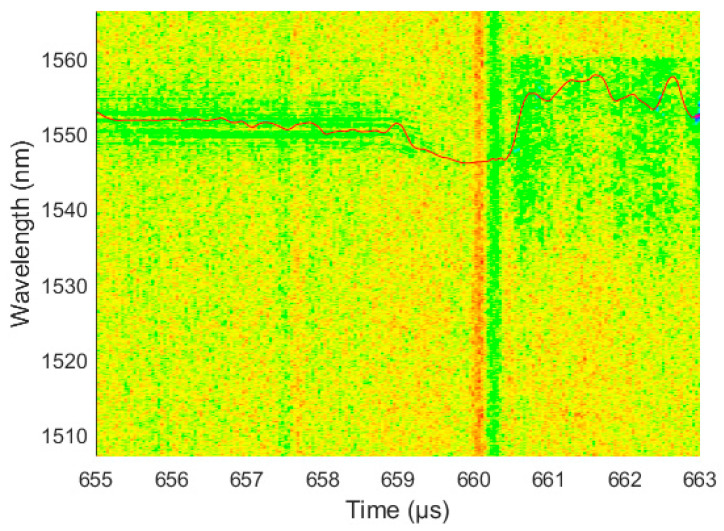
Spectrogram of the multimode sapphire FBG in a low-pressure shock experiment in PMMA. The spectrogram is averaged in the time domain by a factor of 4.

The measured spectrogram was not very contrasted; therefore, it was averaged in the time domain by a factor of four. Consequently, the effective repetition is 25 MHz (one measurement every 40 ns). The averaged spectrogram obtained is shown in Figure 13. The FBG has many narrow peaks; we used the MATLAB 2019b function “tfridge” and some smoothing to obtain the red curve in Figure 13. Our hydrodynamic simulations predict a wavelength shift per kbar of about 0.98 kbar/nm, which is much lower than with a silica fiber. This is due to the laser impedance mismatch between PMMA and sapphire. Simulations also predict the presence of a significant precursor.

In the spectrogram shown in Figure 13, the expected precursor is visible and creates a first wavelength shift of approximately −1.3 nm. Between 659 µs and 660.5 µs, the wavelength shift fluctuates around −5 nm. This value is close to the 4.6 nm simulated. After this value, the signal is incoherent. This was our first attempt to record a wavelength shift from a sapphire FBG from a pressure level. As mentioned in Section 3.1, the shock coupling in a silica or sapphire fiber is not that simple. The longitudinal orientation is more convenient for fiber integration into the target but it degrades the fiber response. An orthogonal orientation should be more suitable for the grating, but the fiber can be broken from shear stress at the edge of the target when the shock front passes through.

### 3.5. Dynamic Strain Measurements

In few defense applications can structures be intensely loaded within a very short time by a blast or an explosive directly in contact. New material assemblies are also studied by isentropic compression generated by a high-pulse power generator [31]. Electrical Strain Gauges (ESGs) [6] are limited in bandwidth to a few hundred kHz and are sensitive to electromagnetic perturbations. Fiber Bragg Grating (FBG) sensors are light, compact, immune to electromagnetic environment sensors, and can be multiplexed in wavelength along a single fiber. They have been used for decades to monitor strain in structural health monitoring, but commercial interrogators are typically available at a rate of a few kHz, and very rarely above 100 kHz.

Fibers with many FBGs can be easily embedded in a carbon fiber-reinforced plastic composite structure. In [32], a composite evaluator was first monitored with three FBGs in temperature during the curing process. Then, this composite evaluator was exposed to a blast and strains were dynamically recoded using our dispersive spectrometer. The residual and dynamic strain measurements during blast loading were successfully compared to finite element models.

**Figure 14:**
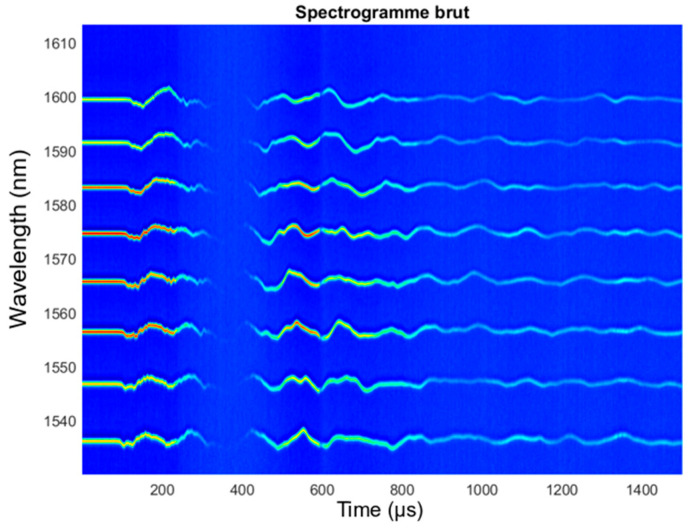
Raw spectrogram of 8 FBGs placed vertically on a cylinder loaded with explosive. The exact wavelength is correct for the first FBG at 1535 nm.

**Figure 15:**
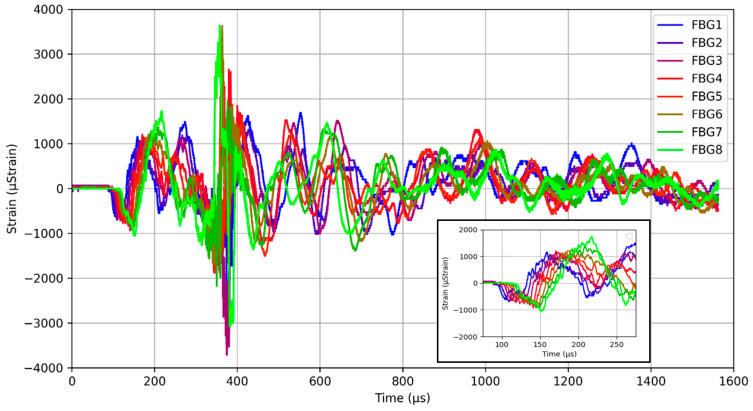
Strain measurements from the 8 FBGs placed on the same single-mode fiber during an explosive loading.

In [33], we presented a first result on dynamic strain measurements on a metallic cylinder asymmetrically loaded with explosives bands using 24 Fiber Bragg Gratings distributed along three fibers. In this paper we present a similar result with eight FBGs placed vertically along a long tube which was loaded with explosive strips from the top to the bottom (Figure 14). In this orientation, the FBGs experience loading one by one. The FBGs are spaced 25 mm apart, so the distance between the first FBG and the eighth is 175 mm. The central wavelengths of the FBGs start at 1536 nm and end at 1592 nm, with a spectral shift of 8 nm between each grating. For the measurement which converts the spectrum in the domain, there is an additional time delay between each FBG of 245 ps. This yields 1.713 ns between the eighth and the first one. The consequence is that in the raw spectrogram shown in Figure 15, only the first FBG has the correct wavelength. The other FBGs are higher in wavelength and will have to be post-processed individually to get the correct wavelength shift. The experiment and the line of eight FBGs were well designed, because the FBGs do not overlap in the spectrogram. Near 380 µs, the spectrogram intensity is lower, and the fiber line between the grating and the measurement system might have encountered additional stress that created optical losses. However, if the spectra are normalized, the wavelength peaks can be tracked.

The wavelength shifts calculated for each FBG are then transposed in microstrains (µstrain) with this coefficient: 1.21 pm/µstrain. The final result is plotted in Figure 15. The measurement lasts 1.562 ms; it is limited by the number of points recorded at 128 Gs/s by the digitizer (20 million per channel). Detonators were used to start the experiment, and the high voltage necessary for their ignition creates parasitic electromagnetic fields. However, thanks to the optical fiber technology, these perturbations have no influence on the results. The first strain is compressive, with a maximum of −1000 µstain with 40 µs for the last FBG (number 8). The curves show delays from this special loading. We can even obtain a good estimate of the detonation velocity by chronometry using the times where the strains start. A value of 6880 m/s is obtained, which is confirmed by other metrology. The maximum strain values measured are +3634 µstrain for FBG8 at 358.7 µs and −3713 µs train for FBG3 at 375 µs.

These measurements were successfully compared to other data (ESPs) and simulations, and it validates the use of the dispersive spectrometer for a chain of FBGs. The measurement system could be adapted to reduce the recording rate to 20–50 MHz, which should be sufficient. The broad spectrum of the laser source of the dispersive spectrometer could allow the use of up to 10 FBGs if the spectral overlaps are limited. With three measurement channels, 30 measurement points with only three fibers can be envisaged, making the system very practical and valuable.

## 4. Conclusions

In this paper we presented the capabilities of a dynamic dispersive spectrometer coupled to one or many Fiber Bragg Grating sensors to measure deflagration, shock, detonation velocities or strain. These extreme measurements require high-frequency response and a high-speed rate. This spectrometer uses an ultrafast laser to generate a pulsed broad spectrum at 100 MHz. The initial optical pulse is then highly chirped to generate a ramp of wavelengths and therefore scan the FBG sensor as fast as the laser repetition rate. The dynamic dispersive spectrometer has been set up differently than previously reported to measure up to three optical fiber lines and to be more robust to chronometry drifts. The system provides the entire spectrum reflected along a fiber with one Chirped FBG (CFBG) or many FBGs. The spectra are recorded one after the other in the time domain. The raw data are recorded at 128 Gs/s before the time demultiplexing step, which is done by numerical signal processing. The time delays between discrete FBGs or between the two edges of a long CFBG must be taken into account. At the end, the spectral resolution is excellent, with 1280 points every 10 ns, and the spectrum width (span) can be adjusted by the dispersion stage from ~20 to ~100 nm.

The different experiments have shown that the FBG sensor response is compatible with the 100 MHz rate. The 3D spectrograms obtained after signal processing are very rich, and complex phenomena can be better understood. For instance, a low-pressure shock wave, which does not damage the silica fiber, can be tracked efficiently within the target. With more modeling, the pressure level could even be evaluated. In explosive physics, phenomena like shock-to-detonation and deflagration-to-detonation are still much studied in order to be properly simulated. Two interesting examples have been presented. A deflagration-to-detonation experiment is particularly difficult to characterize and the long CFBG provided an estimate of the timing of both the compression and reactive phases. An initial pressure measurement from a sapphire fiber was presented. Sapphire has the potential to measure pressure levels up to 200 kbar. FBGs are commonly used to measure strain for structural health monitoring of buildings, bridges, etc. Here, a dynamic experiment of a cylinder loaded with explosives was presented, with eight FBGs spaced apart by 25 mm. A similar system with a slightly reduced recording rate could be created to better suit dynamic strain measurements. The recording rate could also be decreased, which would significantly reduce the cost.

Optical fibers are versatile enough to conceive new types of dynamic experiments. Long CFBGs could be placed on the surface of a complex explosive setup. They also could be placed inside an explosive setup, but manufacturing a narrow hole limits the depth. An alternative is to slice the setup and glue it with the thin fiber in between. It is commonly done for electromagnetic gauges. Regarding the strain measurements, various integrations with multiple FBGs have been validated in many civil applications. The main benefit of the dispersive spectrometer is really the higher recording rate to evaluate the dynamic loading of any structure. The chain of FBGs should be carefully designed to take into account the time delays between gratings so it fits within 10 ns. The central wavelength of the gratings and the dispersion coefficient of the system selected give, in most cases, enough degrees of freedom.

One aspect is that the measurement system remains large, with many discrete components. It would be interesting to make it more compact if more measurement channels are required. It is theoretically possible to integrate the dispersion stage into the mode-locked laser. Instead of optimizing the laser cavity to compress the pulse [34], the dispersion can be managed in the opposite way to obtain highly chirped pulses. Similarly, a mode-locked laser integrated on a chip with a long gain region demonstrated highly chirped pulses with a 4 nm bandwidth at 15 GHz, with reasonable jitter [35]. With more engineering work, a 0.5 GHz integrated chirped source with a spectrum width of at least 30 nm should be possible.

## Data Availability

The raw data supporting this article can be made available by the authors if a reasonable request is made.

## Abbreviations

The following abbreviations are used in this manuscript:

CEA	Commissariat à l’énergie atomique et aux énergies alternatives
CFBG	Chirped Fiber Bragg Grating
DDT	Deflagration-to-Detonation Transition
EDFA	Erbium-doped Fiber Amplifier
EOS	Equations of States
ESG	Electrical Strain Gauges
ESP	Electrical Short Pins
FBG	Fiber Bragg Grating
HMX	High Melting point eXplosive (Octogen)
LANL	Los Alamos National Laboratory
PMMA	Poly(methyl-methacrylate)
SDT	Shock-to-Detonation Transition
